# Editorial of the special issue: vorticity, rotation and symmetry (V)—global results and nonlocal phenomena

**DOI:** 10.1007/s41808-021-00132-x

**Published:** 2021-11-09

**Authors:** R. Danchin, R. Farwig, Š. Nečasová, J. Neustupa

**Affiliations:** 1 Paris, France; 2 Darmstadt, Germany; 3 Prague, Czech Republic

This special issue covers the proceedings of a conference entitled *Vorticity, Rotation and Symmetry (V)—Global Results and Nonlocal Phenomena.* It is the fifth conference in a series of international conferences on mathematical fluid mechanics held at *Centre International de Rencontres Mathématiques (CIRM) in Luminy (Marseille, France)*. This conference was scheduled for April 6–10, 2020, for about 70 participants but had to be cancelled due to the COVID-19 pandemic. We were very glad to have the chance to organize a hybrid conference in the last week of October 26–30, 2020, with about 30 speakers, either on site in Luminy or connected by video conference to a larger international community.

The conference series deals with a lot of recent topics in mathematical fluid mechanics such as the classical questions of existence, regularity and uniqueness as well as asymptotic structure and decay of solutions in time and space of the Euler and Navier–Stokes systems. Further topics are active scalar equations related to fluid mechanics, complex fluids, mixtures, flocking and aggregation, dynamical systems approaches, stabilizing and destabilizing effects, influence by nonlocal terms, free surface flows, and problems of fluid-structure interaction.

This volume contains 15 selected research articles and survey papers of participants of this conference and their coauthors. Classical questions on Navier–Stokes equations are discussed in the papers by M. Beneš, P. Kučera, P. Vacková focussing on various types of boundary conditions in the two-dimensional instationary case, whereas E. Miller wrote a survey of geometric constraints on the possible blowup of three-dimensional solutions in exploiting components in arbitrary (*x*, *t*)-depending directions of the velocity gradient and of the vorticity. A generalized Stokes operator with a bounded measurable matrix-valued coefficient of viscosity and its semigroup is estimated by P. Tolksdorf in local $L^2$-norms by referring to Morrey type estimates of the resolvent. M. Hillairet compares stationary solutions of the Stokes problem in a three-dimensional bounded domain with *N* well-separated holes with solutions of a related Stokes-Brinkman system when the radius of the holes ranges from 1/*N* to the third root of 1/*N*. Recent endpoint regularity estimates in $L^1$ of the heat equation with application to a free boundary value problem of the incompressible Navier–Stokes system near the half-space are summarized by T. Ogawa and S. Shimizu. Two papers deal with questions of magnetohydrodynamics. On one hand, D. Cobb and F. Fanelli stress the role of the velocity in symmetry breaking in ideal incompressible MHD using a Besov space functional framework and the Littlewood-Paley theory. On the other hand, S. Monniaux focuses on the difficulties of MHD equations in bounded domains with Lipschitz boundary and proves the existence of mild solutions.

Several papers deal with compressible fluids. V. Mácha considers the setting of a container with a time-depending moving cavity $\Omega (t)$ and filled by a compressible fluid. Exciting the container by an elastic spring, the author proves the existence of a weak solution. A problem of homogenization of the instationary compressible Navier–Stokes–Fourier system is analyzed by M. Pokorný and E. Skříšovský, therein treating also the entropy inequality and constructing renormalized weak solutions. B. Jin, Y.-S. Kwon, Š. Nečasová and A. Novotný focus on as weak as possible solutions called dissipative turbulent solutions for a mixture of two non-interacting compressible fluids filling a bounded domain with general nonzero inflow/outflow boundary conditions and prove among other things existence of such solutions and a weak strong uniqueness principle in this class. Using the language of Besov spaces, T. Iwabuchi and T. Ogawa survey recent results on ill-posedness for the Cauchy problem of the two-dimensional compressible Navier–Stokes equations for an ideal gas; actually, there exists a sequence of initial data converging to zero and a sequence of epochs $(T_N)$ such that the temperature blows up in certain norms as $T_N\rightarrow 0$.

Another important topic is fluid structure interaction. H. Al Baba, A. Ghosh, B. Muha and Š. Nečasová construct $L^p$-strong solutions to fluid-rigid body interaction systems with Navier slip boundary condition for Newtonian and non-Newtonian fluids. The approach is based on the $\mathcal R$-boundedness and hence maximal regularity of the underlying Stokes operator. Moreover, R. Farwig and A. Schmidt consider in an $L^2$-setting global solutions of viscous flow in the half space $\mathbb {R}^n_+$ with an undamped Kirchhoff-type plate equation for the free elastic surface. The main difficulties compared to related results in the literature are the unboundedness of the domain and the lack of any damping term so that maximal regularity properties for this parabolic–hyperbolic system cannot be used.

A.-L. Dalibard and Ch. Perrin investigate a one-dimensional compressible Navier–Stokes system such that the reciprocal density $v=1/\rho$ satisfies $v\ge 1$ and $(v-1)p=0$. The motivation for this system is given by the modeling of partially congested (or saturated) flows like crowd motions or traffic flows, mixtures or partially pressurized free surface flows. Y. Maekawa investigates the validity of the Prandtl boundary layer expansion for two dimensional Navier–Stokes flows around the Rayleigh boundary layer; he shows stability of the formation of the boundary layer in the inviscid limit with respect to perturbations in the Gevrey 3/2 class.

We would like to express our deep thanks to the administration and staff of CIRM in Luminy for their great support. Without their enormous help and encouragement this hybrid conference would not have been possible. Moreover, we appreciate very much the help of Prof. Michel Chipot, editor-in-chief of this journal, for his willingness to accept and publish the proceedings.

Raphaël Danchin (Paris)

Reinhard Farwig (Darmstadt) 

Šárka Nečasová (Prague) 

Jiří Neustupa (Prague) 

